# Validity of the Norwegian Version of the Needs and Provision Complexity Scale (NPCS) in Patients with Traumatic Brain Injury and Atraumatic Subarachnoid Hemorrhage

**DOI:** 10.3390/jcm13030752

**Published:** 2024-01-28

**Authors:** Marit V. Forslund, Ida M. H. Borgen, Tanja Karic, Ingerid Kleffelgård, Solveig L. Hauger, Marianne Løvstad, Marleen R. van Walsem, Emilie I. Howe, Cathrine Brunborg, Nada Andelic, Cecilie Røe

**Affiliations:** 1 Department of Physical Medicine and Rehabilitation, Oslo University Hospital, 0424 Oslo, Norway; idmbor@ous-hf.no (I.M.H.B.); takari@ous-hf.no (T.K.); uxinff@ous-hf.no (I.K.); solveig.laegreidhauger@sunnaas.no (S.L.H.); emihow@ous-hf.no (E.I.H.); nadand@ous-hf.no (N.A.); cecilie.roe@medisin.uio.no (C.R.); 2 Department of Research, Sunnaas Rehabilitation Hospital, 1453 Bjørnemyr, Norway; marianne.lovstad@sunnaas.no; 3 Department of Psychology, Faculty of Social Sciences, University of Oslo, 0316 Oslo, Norway; 4 Department of Neurohabilitation, Oslo University Hospital, 0424 Oslo, Norway; mwalsem@ous-hf.no; 5 Center for Habilitation and Rehabilitation Models and Services (CHARM), Institute of Health and Society, Faculty of Medicine, University of Oslo, 0316 Oslo, Norway; 6 Centre for Biostatistics and Epidemiology, Oslo University Hospital, 0424 Oslo, Norway; uxbruc@ous-hf.no; 7 Institute of Clinical Medicine, Faculty of Medicine, University of Oslo, 0316 Oslo, Norway

**Keywords:** Needs and Provision Complexity Scale, NPCS, rehabilitation, health services, neurological disorders, brain injury, psychometry, reliability, validity, questionnaire

## Abstract

There is a lack of validated measures in Scandinavian languages to track healthcare service needs and delivery for patients with neurological disabilities. The aim of the present study was to validate the Norwegian version of the clinician and patient Needs and Provision Complexity Scale (NPCS) Needs and Gets. Data on the NPCS from 60 adult patients with traumatic brain injury or atraumatic subarachnoid hemorrhage and symptoms lasting >5 months were assessed for inter-rater/test–retest reliability and agreement, as well as concurrent validity with the Neurological Impairment Scale (NIS), the Functional Independence Measure (FIM), and the Community Integration Questionnaire (CIQ). The clinician NPCS showed good–excellent inter-rater reliability, and the patient NPCS demonstrated good–excellent test–retest reliability. Absolute agreement was moderate–excellent across all clinician and patient items. Concurrent validity was significant, with large correlations between clinician NPCS-Needs and the NIS and FIM total scores, and small–medium correlations between the clinician and patient NPCS-Gets and the NIS and FIM total scores. There were no significant correlations between the NPCS and the CIQ. The study findings support the use of the Norwegian version of the NPCS to assess met and unmet healthcare and support needs for Norwegian-speaking adults with neurological disabilities.

## 1. Introduction

Neurological disorders are the leading cause of disability worldwide and the second leading cause of death [1], showing patterns of increasing global burden over time [2]. Many neurological conditions, including traumatic brain injury (TBI), stroke, Parkinson’s disease, multiple sclerosis, and Huntington’s disease, have debilitating and long-term consequences for patients and their families. Neurological conditions may vary from being stable to progressive, and their impact on an individuals’ needs may vary over time. The healthcare service provision and support should therefore be dynamic to reflect the individuals’ specific needs over time throughout their care pathways.

Until recently, there has been a lack of standardized methods for documenting the patients’ outpatient or community-based healthcare and rehabilitation service needs and delivery [3,4]. The Needs and Provision Complexity Scale (NPCS) was developed in the UK for patients with long-term neurologic disabilities and was published in 2010 [3,5]. The NPCS measures both the needs an individual has for rehabilitation services and support and the extent to which those needs are met through service and support provision. The clinician version consists of two parts, the NPCS-Needs and NPCS-Gets, whereas the patient/caregiver version includes NPCS-Gets. By subtracting NPCS-Needs scores from NPCS-Gets scores, the scale provides a measure of the “degree that the patient needs are met”. The NPCS is relevant on both the individual and the societal level, and an algorithm has been developed to estimate the implications of met and unmet needs in terms of costs [6]. 

There is, however, still scarce information about the validity of the NPCS. The first evaluation of the NPCS psychometrics from the UK [5,7,8] assessed the internal consistency and factor structure of the clinician NPCS-Needs and the patient NPCS-Gets, as well as the test–retest reliability and concurrent validity of the patient NPCS-Gets. There are no studies on the inter-rater reliability and concurrent validity of the clinician NPCS-Needs and NPCS-Gets. 

The NPCS has previously been translated into Norwegian [9], although not validated, and has been used for patients with Huntington’s disease and myotonic dystrophy to assess unmet needs [9,10,11]. The overall objective of the present study was to validate the authorized translated Norwegian clinician and patient version of the NPCS in a sample of patients with persistent symptoms after TBI or atraumatic subarachnoid hemorrhage (aSAH) by adhering to the methodological standards of the consensus-based standards for the selection of health measurement instruments (COSMIN) checklist [12,13,14]. The specific aims were threefold;

(1) To assess the inter-rater agreement and reliability of the clinician NPCS;
(2) To assess test–retest agreement and reliability of the patient NPCS;
(3) To assess concurrent validity of the clinician and patient NPCS with three measures of related constructs.

As there are no prior reports of the inter-rater reliability for the clinician NPCS, we have not made any specific hypothesis regarding the level of inter-rater reliability for the clinician NPCS-Gets and NPCS-Needs. We expected moderate to good test–retest reliability for the patient NPCS-Gets based on the results from psychometric testing of the patient NPCS-Gets in a UK population of patients with different neurological conditions [5]. We hypothesized a medium-sized correlation between the total scores of the NPCS-Gets/-Needs and measures of neurological impairment, independence in activities in daily living, and community integration, and we expected that larger neurological impairment, less independence, and less community integration would correlate to higher service needs and delivery. The hypotheses for concurrent validity were also based on the results of the patient NPCS-Gets in the aforementioned UK study [5].

## 2. Materials and Methods

### 2.1. Study Design and Study Sample

In this cross-sectional study, the participants were recruited through convenience sampling among patients with an ICD-10 diagnosis of TBI (S06.0–S06.9) or aSAH (I60.0–I60.9) who were referred to a specialized head injury outpatient clinic at Oslo University Hospital (OUH). Inclusion lasted from October 2017 to June 2019, and stopped after including *n* = 35 patients with TBI and *n* = 25 patients with aSAH. The inclusion criteria were age 16–85 years at the time of inclusion, symptoms lasting more than five months after injury, and being able to provide informed consent. Exclusion criteria were previously diagnosed severe psychiatric condition or insufficient fluency in the Norwegian language to fill out questionnaires. 

### 2.2. Measures

The main outcome measure of this study is the Needs and Provision Complexity Scale (NPCS) [3,5]. The NPCS is a 15-item measure and consists of two parts: (1) the NPCS-Needs, as rated by clinicians (clinician version); and (2) the NPCS-Gets, as rated by clinicians (clinician version), patients (patient version), or carers (caregiver version). The NPCS has five subscales: healthcare (score range 0–6), personal care (0–10), rehabilitation (0–9), social/family support (0–13), and environment (0–12). Higher scores indicate greater needs or more received services. The internal consistency has been shown to be high, with a Cronbach’s alpha of 0.94, for the full clinician NPCS-Needs; and acceptable, with an alpha of 0.75, for the full patient NPCS-Gets [5]. The internal consistency of the clinician NPCS-Gets scale has not previously been reported.

This study only applied the NPCS clinician and patient version. The clinician version has a total score range of 0–50. Patients’ unmet needs were calculated by subtracting the estimated NPCS-Needs score from the NPCS-Gets score. The NPCS patient version mirrors the clinician version and was scored in the same manner. However, the patient version has a total score range of 0–49 due to different wording resulting in one fewer response category (the patient version lacks the S3 category of social work and case management). 

The “NPCS English version 6 extended” was translated to Norwegian in a previous study [9]. Based on experiences with the questionnaire from the previous project and cultural differences in health service organization between the UK and Norway, an expert consensus group (authors M.V.F., I.M.H.B., T.K., M.R.v.W., E.I.H., N.A., and C.R.) made some minor linguistic adjustments in the Norwegian version 2.0 to better fit the Norwegian healthcare system before validation. None of the described adjustments change the content or the scoring of the questionnaires. The English NPCS version 6 extended (for clinicians) and the Norwegian NPCS clinician and patient version 2.0 is included in the Supplementary Materials.

As there is no current “gold standard” for measuring met and unmet needs, the study explored concurrent validity of the NPCS through correlation with a cross-sectional assessment of impairment level, independence, and community integration with the following scales:

- The Neurological Impairment Scale (NIS) [15]: The NIS is a 17-item measure of neurological impairment (score range 0–50, best to worst). The NIS contains physical (10 items, range 0–29) and cognitive (7 items, 0–21) domains. The full NIS has shown excellent inter-rater reliability between two medical doctors and an internal consistency of 0.75 [15].
- The Functional Independence Measure (FIM) [16,17]: The FIM is an 18-item measure for assessing independence in activities in daily living (score range 18–126, worst to best). The FIM also has two domains: the FIM motor (13 items, range 13–91) and the FIM cognitive (5 items, 5–35) domains. The full FIM has been documented to have excellent reliability with test–retest, interrater reliability, and internal consistency well above 0.90 [18].
- The Community Integration Questionnaire (CIQ) [17,19]: The CIQ is a 15-item scale for assessing the level of participation and community integration (score range 0–29, worst to best). The CIQ has three domains: the home integration (5 items, range 0–10), the social integration (6 items, 0–12), and the productivity (4 items, 0–7) domains. The full CIQ has reported a test–retest reliability from 0.81 to 0.91, an interrater reliability of 0.69, and an internal consistency of 0.76–0.84 in patients with TBI [20].

These three constructs were used for assessing concurrent validity of the NPCS in the UK study [5] and were chosen in this study for the purpose of comparison.

### 2.3. Procedure

Eligible patients were invited by phone to partake in this study prior to the outpatient appointment, and willing participants provided written informed consent at the appointment. Sociodemographic data were collected at the consultation, as were data on symptom burden (Rivermead Post-Concussion Symptoms Questionnaire, RPQ) [21], depressive symptoms (Patients Health Questionnaire, PHQ-9) [22], and anxiety-related symptoms (Generalized Anxiety Disorder scale, GAD-7) [23]. The injury severity, as assessed by acute Glasgow Coma Scale (GCS) score [24], was collected from the medical records. The study participants filled out the NPCS patient version and the CIQ at the consultation (time point 1). For the first patient rating, we gave some general information on how to fill out the NPCS and were available for questions/guidance when patients were filling out the questionnaire. The medical doctor examined the patients neurologically and scored the NIS, whereas the FIM was scored by a certified FIM rater (authors N.A. or I.K.) during the consultation. The patient-reported outcomes and clinical assessment provided the clinicians with information concerning the patient’s needs. Two independent raters filled out the NPCS clinician version separately for each participant during a joint consultation; a medical doctor (author M.V.F. or T.K.) and an interdisciplinary team member—either a psychologist (author I.M.H.B.), neuropsychologist (author S.L.H.), or a physiotherapist (author I.K.). The clinicians had a consensus document describing how to rate the NPCS items based on experiences from the project on Huntington’s disease [9], and this document was updated on the way to simplify the filling out of the clinician NPCS, e.g., with more examples of types of equipment and appropriate scoring categories. The participants were given instructions to complete the NPCS patient version once more after 7 days (time point 2) and return the dated questionnaire via the postal service. The second patient rating was conducted without aid. 

### 2.4. Statistical Analyses

All statistical analyses were conducted in IBM SPSS Statistics version 29 [25]. Descriptive statistics were used to describe the sample characteristics for the total population as well as the subpopulations with TBI and aSAH. The level of significance was set at *p* ≤ 0.05. For group comparison of the two subpopulations, an independent samples T-test was run for normally distributed continuous variables, whereas the Mann–Whitney U-test was used for skewed or non-normally distributed continuous variables, and the Chi-square test was used for categorical variables. The properties of the clinician NPCS-Needs and the clinician and patient NPCS-Gets were examined at the item level for missing data and floor effects (i.e., the lowest score on the NPCS items corresponding to no need or no service provision). The internal consistency of the clinician and patient NPCS could unfortunately not be examined with Cronbach’s alpha or McDonald’s Omega due to a violation of the assumptions of the methods, with zero or very low variance in several of the items. Low variability in responses excluded the possibility of using weighted kappas for reliability assessment. Instead, we examined the proportion of absolute agreement between the two raters for each item through crosstabs, where total agreement was calculated as the number of agreements divided by the total number. We assessed reliability with the intraclass correlation coefficient (ICC, two-way mixed, absolute agreement, average measures) with 95% confidence intervals (CI) for the mean NPCS total and subscale scores. Missing data were not imputed, and cases were excluded pairwise in analyses. Regarding interpretation of reliability, we have used the following classification for both absolute agreement and ICC [26]: values less than 0.5 indicate poor reliability, values between 0.5 and 0.75 indicate moderate reliability, values between 0.75 and 0.9 indicate good reliability, and values greater than 0.90 indicate excellent reliability. Concurrent validity was tested through Spearman’s rho correlation between the clinician and patient NPCS total score and the NIS, FIM, and CIQ total and domain scores. Regarding assessment of correlation size, we have used the following classification for interpretation [27]: 0.10–0.29 indicate a small correlation, 0.30–0.49 indicate a medium correlation, and 0.50–1.00 indicate a large correlation.

## 3. Results

### 3.1. Sample Characteristics

Sample characteristics of the whole population and subpopulations with TBI and aSAH are presented in Table 1. The mean age of the total sample was 44.4 (SD 14.1) years, 63.3% were women and 85.0% had a mild injury according to the acute GCS score. The median time since injury was 295 days (IQR 220–578). The NIS mean total score was 4.8 (SD 2.8), and the FIM mean total score was 124.6 (SD 1.7), indicating good neurological functioning and very high levels of independence in activities of daily living. The CIQ mean total score was 19.2 (SD 4.2), indicating reduced levels of community participation. Regarding symptom burden, the RPQ mean total score was 27.0 (SD 14.0), indicating a severe symptom burden (cut-off ≥ 16) [28]. Regarding emotional distress, the sample scored slightly above the cut-off > 10 for clinically significant depressive symptoms, with a PHQ-9 mean total score of 10.3 (SD 5.5) [29]; whereas the sample reported low levels of anxiety-related symptoms, with a GAD-7 mean total score of 5.4 (SD 4.4). 

The mean total and subscale scores of the clinician and patient NPCS from time point 1 are presented in Table 2. The mean total score of the “Gets” of 5.2 (SD 3.0) for the clinician rating and 5.4 (SD 3.0) for the patient rating indicate that the patients received low levels of healthcare provision overall. The patients mainly received service provision in the subscales of healthcare and rehabilitation. The clinicians rated the highest levels of unmet needs in the subscale of rehabilitation in this mixed TBI/aSAH study population. 

### 3.2. The NPCS Clinician Version: Inter-Rater Agreement and Reliability

The inter-rater reliability of the clinician NPCS-Needs and NPCS-Gets was tested using a single rating from two independent raters at time point 1 (*n* = 60), and there were no missing data. The proportion of absolute agreement between the two healthcare professionals and the response distribution for all items in the NPCS-Needs and the NPCS-Gets are presented in Table 3. The agreement ranges from moderate to excellent across the “Needs” items, and excellent across all “Gets” items. When examining the response distribution, 12/15 (80%) of the items on both the “Needs” and the “Gets” display floor effects, with the majority of responses being in the 0 category, stipulating no assessed need or no service provision, respectively. 

Regarding inter-rater reliability, the ICC for the mean total score of the clinician NPCS-Needs was 0.911 (95% CI 0.847–0.947) and 0.987 (95% CI 0.978–0.992) for the NPCS-Gets (see Table 4). This indicates good to excellent reliability for the clinician NPCS-Needs and excellent reliability for the clinician NPCS-Gets. Across the subscales for both parts, the ICC ranges from moderate to excellent.

### 3.3. The NPCS Patient Version: Test–Retest Agreement and Reliability

Test–retest reliability of the NPCS patient version was conducted with two ratings from each participant reported at a 6–15 days interval, which was considered to be within an appropriate interim period to prevent recall and ensure that the patients remained stable [14]. The level of missing data for the patient NPCS-Gets at time point 2 is shown per item in Table 5 and per subscale and total score in Table 6. The absolute agreement for the test–retest ratings and the response distribution for all items are presented in Table 5 and show that the agreement ranges from moderate to excellent. As with the clinician NPCS, 12/15 (80%) items display floor effects. 

Regarding test–retest reliability (*n* = 43), the ICC for the mean total score of patient NPCS-Gets was 0.867 (95% CI 0.754–0.928) (see Table 6), indicating good to excellent reliability. Across the subscale scores, the ICC mostly ranges from moderate to excellent, with the exception of the social and family support subscale, where the confidence interval places the ICC somewhere between poor and good. 

### 3.4. The NPCS Clinician and Patient Version: Concurrent Validity

Concurrent validity for the clinician and patient NPCS and the other outcomes is shown in Table 7. The correlation between the NPCS clinician rating 1 (MD) and 2 (interdisciplinary team member), measured with Spearman’s rho, was 0.98 for NPCS-Gets and 0.82 for NPCS-Needs. Hence, concurrent validity was only tested for rating 1 of the clinician NPCS-Gets and NPCS-Needs. There were large and significant correlations between the clinician NPCS-Needs and the NIS and FIM total scores, meaning that the larger the neurological impairment or the lower the independence in daily activities, respectively, the larger the assessed need for healthcare and rehabilitation services. Similarly, there was a significant—although small—correlation between the clinician and patient NPCS-Gets and the NIS, and a significant medium correlation with the FIM total scores. Regarding concurrent validity with domain scores, the clinician NPCS-Needs showed significant correlations with all the NIS and FIM domains, varying from medium correlation with both the NIS domains to a small correlation with the FIM motor domain and a large correlation with the FIM cognitive. Both the patient and clinician NPCS-Gets showed small correlations (without significance) with the NIS cognitive domain, and the clinician NPCS-Gets showed a weaker correlation with the FIM motor domain than the other NPCS parts. The correlations between the clinician NPCS-Needs and the CIQ total score and social and productivity domains were small, with the CIQ social domain showing the highest correlation. However, the correlation with the CIQ total and domain scores was not significant for any of the NPCS versions. 

## 4. Discussion

The present study aimed to validate the Norwegian translation of the NPCS clinician and patient version 2.0 in a population of individuals with lasting symptoms after TBI/aSAH. 

In this first report on the inter-rater reliability of the clinician NPCS, the reliability was good to excellent for the NPCS-Needs total score and excellent for that of the NPCS-Gets. The subscale scores, similarly, seem to have adequate reliability. Broken down to the item level, the absolute inter-rater agreement between clinicians ranged from 72 to 100% for the NPCS-Needs (i.e., moderate to excellent), and from 95 to 100% for the NPCS-Gets (i.e., excellent), but note that this approach does not consider random error. Responses from the clinicians regarding the assessment of patient needs could be dependent on clinical background and professional field. We included several different interdisciplinary team members as the second rater in order to mimic clinical practice in the study design. We anticipated that the reliability would tend to be higher for the NPCS-Gets than the NPCS-Needs as the NPCS-Gets is merely a report of what type of services and support the patient receives, whereas the NPCS-Needs depends, to a larger degree, upon clinical judgement. However, such a high level of reliability for the clinician NPCS-Needs was a bit surprising and might suggest that the variability within responses has been reduced due to the floor effects observed in the sample. 

The expected moderate to good test–retest reliability for the patient NPCS-Gets was partly confirmed, as the results showed good to excellent reliability for the NPCS-Gets total score. The social and family support subscale diverged from the rest, with poor to good reliability, and was the subscale with the lowest item variance of the NPCS-Gets. At the item level, the test–retest absolute agreement ranged from 74% to 100% (i.e., moderate to excellent). To compare with the UK validation study [5], the authors reported ICCs ranging from 0.65 to 0.84 for patient NPCS-Gets subscales, indicating moderate to good reliability. The largest differences were within the rehabilitation subscale, with an ICC of 0.65 in their study compared to an ICC of 0.84 in our study; and within the healthcare subscale, with an ICC of 0.67 compared to an ICC of 0.83 in our study. On the patient NPCS-Gets item level, they reported absolute agreement ranging from 60% to 95%. 

We hypothesized medium-sized concurrent correlations for both the NPCS-Gets and -Needs but found varying levels of correlation—from small to large—for the NIS and FIM, whereas the correlation with the CIQ total score only reached small for NPCS-Needs. The correlations, in general, were the largest for the clinician NPCS-Needs regarding the NIS and the FIM, with all correlations reaching significance. The patient and clinician NPCS-Gets also showed significant correlations with the NIS and the FIM total score, but with lower strength of relationship and more mixed results at the domain level. The clinician NPCS-Unmet Needs total score was calculated to a mean of 1.1 in the present patient population, indicating unmet needs. As the NIS and the FIM measures neurological impairment and functioning, respectively, the populations’ unmet needs can explain the differences in the size of correlation for the NPCS-Needs and -Gets. The correlations with the CIQ total score and domains were not significant for either of the NPCS versions. As the CIQ measures participation within different life domains, it was surprising that the strength of the relationship approached zero for the NPCS-Gets versions, as if there was no relationship at all between the variables. The concurrent validity results in our study were somewhat different from the UK validation study [5], especially regarding the CIQ. Siegert et al. assessed the concurrent validity of the patient NPCS-Gets domain scores and found significant correlation with all measures’ total scores. In their study, the correlation with the NIS total score ranged from 0.27 to 0.47, and with the CIQ total score from 0.39 to 0.44. For functional independence, they used measures other than those in our study, i.e., the Barthel Index and the Northwick Park Nursing Dependency Scale, with reported effect sizes for total scores ranging from 0.54 to 0.65, which was higher than we found for the FIM total score. The UK study had patients of mixed etiology that had all been hospitalized due to their neurological condition or injury (stroke/SAH 51.7% and TBI 13.3%) and had a higher mean NIS score of 12.7 at six months after discharge (i.e., the time of rating), indicating larger neurological disabilities in their population. In our sample of mostly mild brain injuries and longer time since injury, one possibility is that the reduced community integration in our population may have a more multifactorial and biopsychosocial explanation.

The NPCS patient version uses some phrases and terminology regarding the healthcare system that the patients sometimes struggled to understand, such as the meaning of help for community-based activities, social work and case management, and advocacy needs. One recurring issue was that the patients often became confused about the difference between services to be reported under the section on medical care needs and the section of therapy needs (i.e., doctor follow-up vs. rehabilitation follow-up). The level of support provided by clinicians and their being available for questions during the first patient rating was considered useful. 

Although not formally assessed, the feedback from the healthcare personnel raters was that the questionnaire was easily understood for most items, but that the vocational/educational support had some overlap in categories, and that the specialist equipment item was hard to rate without knowledge on the complexity of the specific types of equipment. The consensus document from the previous project was thus considered valuable [9], especially regarding concrete examples of equipment. In practice, uncertainty about the scoring level of an item was solved by describing the patient’s situation or equipment in the margin of the questionnaire and then asking someone with broader knowledge on the subject for clarification before giving a final response. For example, one could ask an occupational therapist or physician about types of equipment, a physician about medical follow-up regimes, or a social worker about the content of different types of vocational support programs.

The NPCS is the first validated questionnaire for documenting patients’ outpatient and community-based service needs and delivery in a Scandinavian language. All the Scandinavian countries are welfare countries with similarly organized publicly funded healthcare systems with universal access. The patient populations are comparable across these countries regarding their sociodemographic and cultural background, suggesting that a common Scandinavian assessment tool such as the NPCS would be appropriate. The availability of a validated scale that is simple to use and efficient in demonstrating patients’ unmet needs is highly relevant, as research has reported unmet service needs in both the subacute and long-term phase following acquired brain injury [30,31,32]. 

### Limitations and Future Directions

The NPCS was developed for individuals with long-term neurological conditions to offer a standardized way to assess healthcare service provision and unmet needs through different stages of patient care pathways. We used a convenience sample from our outpatient clinic and, based on clinical experience, expected variation in impairment and service provision, especially within the subpopulation of individuals with aSAH. However, although the included population reported severe symptom burden, they turned out to have low neurological impairment and high functional independence, resulting in clear floor effects in several items and subscales of the NPCS as reported by both clinicians and patients. The low spread in NPCS categories proposed limitations with regards to statistical methods with which to evaluate reliability and internal consistency appropriately and, e.g., excluded the possibility of using weighted kappas, Cronbach’s alpha, or McDonald’s Omega. On the other hand, the internal consistency has already been evaluated for the English version and was thus considered less important in the present validation, despite being recommended by the COSMIN checklist. For all three assessed NPCS parts, the items with zero variance were within the social and family support subscale.

The NPCS questionnaire may be reliable for use in this TBI/aSAH population, but its suitability for use in patient populations with high independence levels may be questioned. The most relevant subscales of the NPCS-Needs/-Gets were the healthcare and rehabilitation subscales, and for some, the item on specialist equipment under the environment subscale. For patients with more severe or progressive neurological conditions and complex needs, the questionnaire will probably be more appropriate to use with all subscales. As the questionnaire uses terminology that patients may struggle to understand, we advise clinicians to aid the patients in filling out the questionnaire. 

The NPCS validation in this study adheres to the methodological standards proposed in the COSMIN checklist, such as the inter-rater/test–retest reliability, concurrent validity, and hypotheses testing. However, other measurement properties, such as the predictive validity and responsiveness, were beyond the scope of this study, despite being on the COSMIN checklist. The Norwegian NPCS version 2.0 is included as an outcome measurement in two larger research projects on populations with traumatic brain injury and multitrauma [33,34], so further Norwegian publications on unmet needs using the NPCS are expected in the foreseeable future.

## 5. Conclusions

The study results indicate that the Norwegian clinician version of the NPCS has good to excellent inter-rater reliability and that the patient version has good to excellent test–retest reliability. The absolute agreement was moderate–excellent across the clinician and patient NPCS items. Concurrent validity was significant, with large correlations between the clinician NPCS-Needs and the NIS and the FIM total scores; whereas the correlation was small to medium for the NIS and the FIM total scores for both the clinician and patient NPCS-Gets. The validity testing of the Norwegian version of the clinician and patient versions shows promising results; however, the statistical methods was somewhat limited due to a population with high functional levels and accompanying floor effects in scores. The study findings support the use of the Norwegian version of the NPCS to assess the met and unmet needs for Norwegian-speaking individuals with neurological disabilities.

## Figures and Tables

**Table 1:** Characteristics of the total study population and subpopulations with traumatic brain injury and atraumatic subarachnoid hemorrhage with group comparison.

	Total Population (*n* = 60)	TBI Population (*n* = 35)	aSAH Population (*n* = 25)	Group Comparison, *p*-value
**Sociodemographic variables**	***n* (%)**	***n* (%)**	***n* (%)**	
Age at follow-up, mean (SD)	44.4 (14.1)	37.7 (10.4)	53.8 (13.5)	*<0.001* ^1^
Sex				0.239 ^2^
Male	22 (36.7%)	15 (42.9%)	7 (28.0%)	
Female	38 (63.3%)	20 (57.1%)	18 (72.0%)	
Marital status				0.083 ^2^
Married, domestic partner	41 (68.3%)	27 (77.1%)	14 (56.0%)	
Single	19 (31.7%)	8 (22.9%)	11 (44.0%)	
**Injury-related variables**	***n* (%)**	***n* (%)**	***n* (%)**	
Severity of injury				
Mild (GCS 13–15)	51 (85%)	33 (94.3%)	18 (72.0%)	NA
Moderate (GCS 9–12)	4 (6.7%)	0 (0.0%)	4 (16.0%)	
Severe (GCS 3–8)	5 (8.3%)	2 (5.7%)	3 (12.0%)	
Days since injury, median (IQR)	295 (220–578)	337 (258–795)	233 (204–414)	*0.020* ^3^
**Functional outcome measures**	**Mean (SD), min–max**	**Mean (SD), min–max**	**Mean (SD), min–max**	
Neurological Impairment Scale				
Total score	4.8 (2.8), 0–12	5.4 (2.2), 0–9	4.0 (3.3), 0–12	*0.030* ^3^
Physical	2.8 (1.6), 0–6	3.2 (1.3), 0–6	2.2 (1.8), 0–6	*0.011* ^3^
Cognitive	2.0 (1.5), 0–6	2.1 (1.4), 0–5	1.8 (1.7), 0–6	0.251 ^3^
Functional Independence Measure				
Total score	124.6 (1.7), 116–126	124.6 (1.2), 121–126	124.5 (2.2), 116–126	0.427 ^3^
Motor	90.9 (0.7), 86–91	91.0 (0.2), 90–91	90.7 (1.1), 86–91	0.155 ^3^
Cognitive	33.7 (1.3), 30–35	33.7 (1.2), 30–35	33.8 (1.5), 30–35	0.321 ^3^
Community Integration Questionnaire				
Total score	19.2 (4.2), 5–28	19.1 (4.0), 12–27	19.4 (4.5), 5–28	0.805 ^1^
Home	6.9 (2.4), 0–10	6.3 (2.2), 0–10	7.7 (2.4), 2–10	*0.017* ^3^
Social	8.9 (2.1), 2–12	9.1 (1.9), 5–12	8.7 (2.5), 2–12	0.727 ^3^
Productivity	3.5 (2.2), 0–7	3.8 (2.3), 0–7	3.0 (1.9), 0–6	0.175 ^3^
**Patient-reported outcome measures**	**Mean (SD), min–max**	**Mean (SD), min–max**	**Mean (SD), min–max**	
Rivermead Post-Concussion Symptoms Questionnaire	27.0 (4.0), 0–56	31.1 (13.5), 0–56	21.2 (12.8), 0–43	*0.006 ^1^*
Patients Health Questionnaire ^4^	10.3 (5.5), 0–26	10.7 (5.3), 0–26	9.7 (5.9), 0–21	0.478 ^1^
Generalized Anxiety Disorder scale ^4^	5.4 (4.4), 0–21	5.7 (3.9), 0–21	5.1 (5.1), 0–18	0.329 ^3^

Significant *p*-values in italic. NA: non-applicable; aSAH: atraumatic subarachnoid hemorrhage; TBI: traumatic brain injury. ^1^ Tested with the Independent Samples T-test. ^2^ Tested with the Chi-Square test. ^3^ Tested with the Mann–Whitney U-test. ^4^ *n* = 24 for the aSAH population.

**Table 2:** The mean total and subscale scores of the NPCS clinician and patient version at the first time point (*n* = 60).

NPCS	Clinician Version ^1^			Patient Version
	“Needs”, Mean (SD)	“Gets”, Mean (SD)	Unmet Needs (Needs–Gets)	“Gets”, Mean (SD)
Total score	6.3 (2.9)	5.2 (3.0)	1.1	5.4 (3.0)
Healthcare	2.2 (0.8)	2.0 (1.1)	0.2	2.0 (1.1)
Personal care	0.3 (0.9)	0.2 (0.8)	0.1	0.3 (0.9)
Rehabilitation	3.5 (1.8)	2.8 (2.0)	0.7	2.7 (2.0)
Social and family support	0.2 (0.6)	0.1 (0.3)	0.1	0.1 (0.4)
Environment	0.3 (0.8)	0.3 (0.8)	0.0	0.2 (0.6)

^1^ Used clinician-rating 1.

**Table 3:** Absolute agreement between the ratings of two independent healthcare professionals and response distribution on items of the NPCS clinician version (*n* = 60).

NPCS Subscales	NPCS Items		NPCS-Needs		NPCS-Gets	
	Items	Possible Score Range	Most Frequent Response, Distribution	Absolute Agreement Needs	Most Frequent Response, Distribution	Absolute Agreement Gets
Healthcare	Medical care	M0–M3	43.3% M2, M0–M3 ^1^	0.72	51.7% M2, M0–M3 ^1^	0.97
	Skilled nursing	N0–N3	91.7% N0, N0–N2	0.95	91.7% N0, N0–N3	0.95
Personal care	Number of carers	CN0–CN2	90% CN0, CN0–CN1	0.98	91.7% CN0, CN0–CN1	0.98
	Care frequency	CF0–CF5	90% CF0, CF0–CF3	0.97	91.7% CF0, CF0–CF3	0.98
	Personal assistant/enabler	PA0–PA3	95% PA0	1.00	96.7% PA0, PA0–PA1	1.00
Rehabilitation	Number of therapy disciplines	TD0–TD3	31.7% TD2, TD0–TD3 ^1^	0.73	38.3% TD1, TD0–TD3 ^2^	0.95
	Therapy intensity	TI0–TI3	35.0% TI2, TI0–TI3 ^1^	0.72	35.0% TI2, TI0–TI3 ^1^	1.00
	Vocational/educational support	VR0–VR3	53% VR0, VR0–VR3	0.72	81.7% VR0, VR0–VR3	1.00
Social and family support	Social work/case management	S0–S3	90% S0, S0–S1	0.98	95% S0, S0–S1	1.00
	Family carer support	FC0–FC3	91.7% FC0, FC0–FC2	0.98	100% FC0	1.00
	Respite—residential	RR0–RR3	100% RR0	1.00	100% RR0	1.00
	Respite—day care	RD0–RD2	100% RD0	1.00	100% RD0	1.00
	Advocacy support	AD0–AD2	98.3% AD0, AD0–AD1	0.98	98.3% AD0, AD0–AD1	0.98
Environment	Equipment	E0–E3	90% E0, E0–E2	1.00	90% E0, E0–E2	1.00
	Accommodation	AC0–AC9	96.7% AC0, AC0 + AC4	0.98	96.7% AC0, AC0 + AC4	0.98

^1^ The responses were well distributed within categories 0–3. ^2^ The responses were mainly distributed within categories 0–2.

**Table 4:** Inter-rater reliability of the clinician NPCS-Needs and NPCS-Gets total and subscale scores assessed with intraclass correlation coefficient (*n* = 60).

NPCS Clinician	NPCS-Needs		NPCS-Gets	
	ICC Average Measure	95% CI Average Measure	ICC Average Measure	95% CI Average Measure
Total score	0.911	0.847–0.947	0.987	0.978–0.992
Healthcare	0.749	0.581–0.850	0.982	0.970–0.989
Personal care	0.971	0.951–0.982	0.973	0.955–0.984
Rehabilitation	0.879	0.798–0.927	0.997	0.995–0.998
Social and family support	0.958	0.930–0.975	0.939	0.899–0.964
Environment	0.850	0.749–0.910	0.850	0.749–0.910

ICC = intraclass correlation coefficient; CI = confidence interval.

**Table 5:** Absolute agreement between the test–retest ratings of the patient and response distribution on items of the patient NPCS-Gets.

NPCS Subscales	NPCS Items		N	Most Frequent Response, Distribution	Absolute Agreement Gets
	Items	Possible Score Range			
Healthcare	Medical care	M0–M3	50	34.0% M2, M0–M3 ^1^	0.74
	Skilled nursing	N0–N3	51	92.2% N0, N0–N3	0.96
Personal care	Number of carers	CN0–CN2	51	88.2% CN0, CN0–CN1	0.94
	Care frequency	CF0–CF5	51	86.3% CF0, CF0–CF3	0.92
	Personal assistant/enabler	PA0–PA3	51	90.2% PA0, PA0–PA1	0.96
Rehabilitation	Number of therapy disciplines	TD0–TD3	50	42.0% TD1, TD0–TD3 ^1^	0.90
	Therapy intensity	TI0–TI3	49	36.7% TI2, TI0–TI3 ^1^	0.80
	Vocational/educational support	VR0–VR3	50	74.0% VR0, VR0–VR3	0.86
Social and family support	Social work/case management	S0–S2	51	92.2% S0, S0–S2	0.94
	Family carer support	FC0–FC3	51	98.0% FC0, FC0 + FC3	0.98
	Respite—residential	RR0–RR3	51	98.0% RR0, RR0 + RR1	0.98
	Respite—day care	RD0–RD2	50	100% RD0	1.00
	Advocacy support	AD0–AD2	49	100% AD0	1.00
Environment	Equipment	E0–E3	50	90.0% E0, E0–E3	0.96
	Accommodation	AC0–AC9	50	92.0% AC0, AC0–AC3	0.96

^1^ The responses were well distributed within categories 0–3.

**Table 6:** Test–retest reliability of the patient NPCS-Gets total and subscale scores assessed with intraclass correlation coefficient.

Patient NPCS-Gets	N	ICC Average Measure	95% CI Average Measure
Total score	43	0.867	0.754–0.928
Healthcare	50	0.832	0.704–0.905
Personal care	51	0.779	0.612–0.874
Rehabilitation	48	0.840	0.715–0.911
Social and family support	48	0.577	0.247–0.762
Environment	49	0.869	0.768–0.926

ICC = intraclass correlation coefficient; CI = confidence interval.

**Table 7:** Correlations between the clinician and patient NPCS total scores and the NIS, FIM, and CIQ total and domain scores with Spearman’s rho (*n* = 60).

	Clinician NPCS-Needs		Clinician NPCS-Gets		Patient NPCS-Gets	
	Rho	Sig.	Rho	Sig.	Rho	Sig.
Neurological Impairment Scale						
Total	0.515	*<0.001*	0.274	*0.034*	0.293	*0.023*
Physical	0.481	*<0.001*	0.387	*0.002*	0.380	*0.003*
Cognitive	0.434	*<0.001*	0.128	0.328	0.174	0.184
Functional Independence Measure						
Total	−0.614	*<0.001*	−0.362	*0.005*	−0.414	*0.001*
Motor	−0.272	*0.036*	−0.135	0.302	−0.282	*0.029*
Cognitive	−0.573	*<0.001*	−0.353	*0.006*	−0.365	*0.004*
Community Integration Questionnaire						
Total	−0.181	0.167	−0.055	0.677	−0.058	0.658
Home	0.076	0.566	0.081	0.540	0.092	0.486
Social	−0.228	0.080	−0.103	0.435	−0.130	0.322
Productivity	−0.199	0.127	−0.107	0.415	−0.097	0.461

Significant *p*-values in italic.

## Data Availability

The dataset analyzed in the present study is not publicly available due to ethical and legal regulations regarding protection of personal data.

## Supplementary Materials

The following supporting information can be downloaded at https://www.mdpi.com/article/10.3390/jcm13030752/s1: Supplementary Material File S1: The Needs and Provision Complexity Scale (NPCS) English version 6 extended for clinicians; Supplementary Material File S2: The Needs and Provision Complexity Scale (NPCS) Norwegian version 2.0 for clinicians; Supplementary Material File S3: The Needs and Provision Complexity Scale (NPCS) Norwegian version 2.0 for patients.
