# Supplementary material for: Validity of the Norwegian Version of the Needs and Provision Complexity Scale (NPCS) in Patients with Traumatic Brain Injury and Atraumatic Subarachnoid Hemorrhage
Source: J Clin Med. 2024 Jan 28;13(3):752. doi: 10.3390/jcm13030752 (PMC10856215; doi:10.3390/jcm13030752)
Supplement: Supplementary file 1 [file jcm-13-00752-s001.zip › Supplementary Material File S2 NPCS_clinician version 2.0_Norwegian.pdf]

KING'S College LONDON

University of London

The North West London Hospitals NHS

NHS Trust

Skala for behovs- og tjenestekompleksitet ved kroniske nevrologiske sykdommer (NPCS)

Utvidet versjon for helsepersonell

Skjemaet kan brukes og kopieres fritt,

men må ha referanse til opphavspersonene ved bruk i publikasjoner

For mer informasjon og råd, ta kontakt med:

Professor Lynne Turner-Stokes DM FRCP
Herbert Dunhill Chair of Rehabilitation, King's College London.

Norsk versjon 2.0 - 2017

Bakgrunn

Det nasjonale tjenesterammeverket (NSF) for kroniske nevrologiske tilstander (LTNC) legger opp til integrerte tjenester som kan gi helhetlig, personsentrert omsorg (Department of Health, 2005).

Dette omfatter 11 kvalitetskrav som dekker omsorgsforløpet fra diagnose til død. Integrert omsorgsplanlegging (Kvalitetskrav 1) utgjør det avgjørende grunnlaget for NSFs anbefalinger.

Det erkjennes imidlertid at det finnes svært begrensede ressurser for integrert omsorgsplanlegging, og dette representerer en stor trussel mot gjennomføringen av NSFs anbefalinger.

Det er derfor avgjørende å være i stand til å vurdere tjenestetilbudet i forhold til behov, både på individuelt nivå og på befolkningsnivå, for å kunne fokusere tjenesteutviklingen på en effektiv måte.

Kroniske nevrologiske tilstander utgjør i seg selv en svært mangfoldig gruppe, og blant disse har folk svært ulike behov for tjenester. Hvorvidt tjenestetilbudet er tilstrekkelig må vurderes opp mot dette.

Diagnose er en dårlig ledetråd til behov i denne sammenheng, og vi trenger en annen metode for å definere behovet for tjenester. Skalaen for behovs- og ytelseskompleksitet (Needs and Provision Complexity Scale – NPCS) er utformet for å måle behov for lokal omsorg og rehabilitering, og vurdere tilbudet opp mot disse behovene.

Oversikt over skalaen for behovs- og omsorgskompleksitet (NPCS)

Skalaen er en ordinalskala med fem hoveddomener og femten underskalaer som er oppsummert i tabellen under. Den har en total poengfordeling fra 0 til 50.

**Domener**	**Inter-vall**	**Punkter**	**kode**	Skårintervall	
				Behov	Mottar
**Helsetjenester**	**0-6**	**Behov for medisinsk omsorg**	**M**	0-3	0-3
		**Behov for fagsykepleie**	**N**	0-3	0-3
**Personlig omsorg**	**0-10**	**Antall omsorgspersoner**	**CN**	0-2	0-2
		**Omsorgsfrekvens**	**CF**	0-5	0-5
		**Personlig assistent / tilrettelegger**	**PA**	0-3	0-3
**Rehabilitering**	**0-9**	**Terapidisipliner**	**TD**	0-3	0-3
		**Terapiintensitet**	**TI**	0-3	0-3
		**Yrkesrettet støtte /rehabilitering**	**VR**	0-3	0-3
**Sosial støtte og**	**0-13**	**Sosial rådgivning og oppfølging**	**S**	0-3	0-3
**familiestøtte**		**Støtte til omsorgspersoner i familien**	**FC**	0-3	0-3
		**Avlastning - i institusjon**	**RR**	0-3	0-3
		**Avlastning - på dagtid**	**RD**	0-2	0-2
		**Verge**	**AD**	0-2	0-2
**Miljø**	**0-12**	**Utstyr**	**E**	0-3	0-3
		**Bolig**	**AC**	0-9	0-9
**Sum**	**0-50**				

NPCS engelsk versjon 6; Turner Stokes et al. NPCS norsk versjon 1.0; Akasie språktjeneste, Frich og van Walsem.
NPCS norsk versjon 2.0; Forslund et al.

NPCS er inndelt i to deler:

Del A: NPCS-Behov – definerer behovet for bistand under de ulike overskriftene (NPCS registrerer både faglige og personlige perspektiver på hva pasienten trenger (faglig syn) og hva de ville ønske (personlig valg).

Del B: NPCS-Mottar (eller **'Tjenester'**) – definerer omfanget av den bistand eller de tjenester som i dag blir gitt i den samme strukturen.

Udekkede behov – blir dermed registrert som forskjellen mellom de to skalaene.

Generelt sett er skåringsnivåene under hvert punkt utformet for å fastslå:
a) antallet personell det er behov for og
b) behovs-/intervensjonshyppighet.
Omfangsbeskrivelsene gir en viss indikasjon på hva som kan være "av og til", "regelmessig" og "ofte", <u>men disse er ikke ment å være strengt definerte grensepunkter.</u>

Det oppsummerende skåringsarket omfatter en sjekkliste for de bestemte tjenestene det er behov for under hver overskrift. Det har også et sett med rammer der man kan registrere årsakene til avvik (udekket behov), som kan være:

a) *Tjenesten er ikke tilgjengelig* – dvs. det finnes ingen tjeneste tilgjengelig eller den er ikke blitt tilbudt
b) *Tjenesten er avslått* – dvs. tjenesten har blitt tilbudt, men avslått av pasienten/ omsorgspersonen (dette vil ofte være fordi de mener at det som er blitt tilbudt ikke passer) – det er plass til å registrere de spesielle omstendigheten om ønskelig.
c) *Annet* - en annen grunn

'Trenger, ønsker og får'

Det er også en mulighet til å skåre 'det pasienten ønsker'.

Dette er spesielt relevant i to scenarioer:

a) der fagpersonalet mener at pasienten trenger en bestemt tjeneste, men pasienten aksepterer den ikke.
Et typisk eksempel kunne være der fagpersonalet mener at en person trenger omsorg for å kunne være under trygg overvåking, men vedkommende nekter dette, enten fordi de mangler innsikt i sine egne problemer, eller fordi de ønsker å opprettholde sin uavhengighet og autonomi.

b) der fagpersonalet mener at pasienten ikke trenger en bestemt tjeneste, fordi det ikke er noe klinisk potensial til å dra nytte av den, men pasienten og/eller familien ønsker tjenesten fordi de føler at den kan være til hjelp.
Et typisk eksempel kunne være en situasjon hvor en person har en permanent fysisk bevegelseshemming som ikke har mulighet til å endre seg ved fysioterapi, men et vedlikeholdsprogram er blitt foreskrevet. Personen og/eller familien holder imidlertid sterkt fast ved troen på at med tilstrekkelig terapi vil de få førligheten tilbake.

I begge disse situasjonene har pasienten/familien et gyldig synspunkt som bør registreres, men av ulike grunner kan de tjenestene de ønsker ikke gis.

Skala for behovs- og tjenestekompleksitet (NPCS) for kroniske nevrologiske sykdommer

PASIENTINFORMASJON		
**Navn:**	**NHS-nr:**	**Dato for skåring:...../...../.......**

DEL A - BEHOV: **For hver underskala, sett en ring rundt <u>det høyeste nivået</u> som passer**

1. BEHOV FOR MEDISINSK OPPFØLGING – behov for intervensjon fra en lege for **utredning, tilsyn eller behandling.**

- Medisinsk spesialistbehandling omfatter <u>alle</u> medisinske spesialiteter.

**M 0**	**Fastlege, av og til –** ingen regelmessig kontakt – oppsøker fastlegen selv etter behov	Type med. oppfølging<br>☐ Nevrologi<br>☐ Rehab. medisin<br>☐ Nevropsykiatri<br>☐ Palliativ medisin<br>☐ Annet<br>- Nevrokirurgi<br>- Ortopedi<br>- Psykiatri
**M 1**	**Fastlege, aktivt tilsyn -** regelmessig tilsyn/behandling, bare hos fastlegen	
**M 2**	**Spesialiststøtte på lavt nivå** f.eks. for hovedsaklig stabil tilstand (f.eks. 1-2 besøk per år)<br><u>Løpende tilsyn/behandling hos FL med råd/vurdering hos spesialist av og til</u>	
**M 3**	**Aktiv intervensjon fra medisinsk spesialist er påkrevet** f.eks. for foranderlig/ ustabil tilstand eller for uavklarte symptomer (f.eks. ≥3 besøk per år)<br>Undersøkelse eller behandling som krever hyppig kontakt med spesialisert medisinsk team	

2: BEHOV FOR SPESIALSYKEPLEIE – **trenger intervensjon fra spesial- og/eller spesialtrent sykepleiepersonale,** f.eks. helsesøster eller spesialsykepleier (f.eks. for sårpleie, håndtering av blære-/ tarmfunksjonen / tilsyn med medisinbruk / spesialisert rådgivning/støtte/veiledning)

**N 0**	**Ikke behov** for spesialsykepleier	Typer sykepleie<br>☐ Hjemmesykepleier<br>☐ Spesialsykepleier<br>☐ Nevrologi<br>☐ Psykiatri<br>☐ Palliativ omsorg<br>☐ Annet<br>- Sårsykepleier<br>- Stomisykepleier
Trenger **intervensjon** fra en **spesialsykepleier/spesialtrent sykepleier:**		
**N 1**	**Intervensjon av og til** (f.eks. **en gang i måneden eller sjeldnere)**	
**N 2**	**Regelmessig intervensjon** (f.eks. **hver eller annenhver uke)**	
**N 3**	**Hyppig intervensjon,** (**hver dag** eller **flere ganger i uken)**	

3. PERSONLIG PLEIE - **I og rundt hjemmet**

3a: Antall pleiere: **Behov for hjelp med <u>grunnleggende egenpleie</u>**		
**CN 0**	**Ingen pleiere** nødvendig for grunnleggende omsorg	Hvem yter denne hjelpen?:<br>☐ Uformell familieomsorg<br>☐ Formelle betalte pleiere<br>☐ Andre
**CN 1**	Behov for **hjelp fra 1 person** for de mest grunnleggende omsorgsbehovene	
**CN 2**	Behov for **hjelp fra ≥2 personer** for de mest grunnleggende omsorgsbehovene	

3b: Omsorgsfrekvens: **Omsorgsfrekvens for hjelp med <u>grunnleggende egenpleie,</u> inkludert trygghet**	
**CF 0**	**Ikke behov** for hjelp med egenpleie.
**CF 1**	**Behov av og til** – sjeldnere enn daglig for hjelp med egenpleie eller utvidede daglige aktiviteter

**CF 2**	Behov for **regelmessig hjelp én gang om dagen**
**CF 3**	Behov for **regelmessig hjelp 2-3 ganger om dagen** – vil kunne dekkes av en periodisk oppsøkende omsorgspakke. Kan være trygt alene i >4 timer og trenger ikke omsorg/tilsyn om natten
**CF 4**	**Hyppige eller uforutsigbare omsorgsbehov**, trenger nærvær av noen det meste av tiden.<br>Kan ikke være trygt alene i >4 timer, eller trenger omsorg/tilsyn om natten (men ikke våkende nattevakt)
**CF 5**	**Trenger konstant tilsyn -** kan ikke være alene i huset, selv i korte perioder<br>OG/ELLER trenger **våkende nattlig omsorg –** trenger > 2 intervensjoner om natten)

3c: Personlig assistent/tilrettelegger **Bistandsfrekvens for deltagelse i lokale aktiviteter på dagtid**

**PA 0**	**Ikke behov** for bistand til lokale aktiviteter	Type:<br>☐ Brukerstyrt assistent<br>☐ Støttekontakt<br>☐ Treningskontakt
**PA 1**	**Behov av og til** – 1-2 dager i uken	
**PA 2**	**Hyppig behov** – 3-5 dager i uken	
**PA 3**	**Daglig** – 6-7 dager i uken	

4. BEHOV FOR BEHANDLING **– inkludert poliklinisk, aktivitetsbasert og yrkesrettet rehabilitering**

Antall faggrupper: - behov for å være aktivt involvert i behandling (dvs. minst 1 time i måneden)

**TD 0**	**0**	**Kryss av påkrevde faggrupper:**	
**TD 1**	**Bare én enkelt faggruppe**	☐ Fysioterapi<br>☐ Ergoterapi<br>☐ Logopedi<br>☐ Dietetiker/ ernæringsfysiolog<br>☐ Ortopedi/protese	☐ Psykologi<br>☐ Familieterapeut<br>☐ Psykiatri<br>☐ Annet:<br>- Sosionom<br>- Optiker
**TD 2**	**Enkeltstående faggrupper,** ikke koordinert		
**TD 3**	**Koordinert tverrfaglig team**		

Behandlingsintensitet: - Generell intensitet i de faglige behandlingstiltakene som det er behov for

**TI 0**	**Ikke behov** for faglige behandlingstiltak
**TI 1**	**Behov for periodevis vurdering eller vedlikeholdsprogram** – **ELLER** trenger **bare gruppeterapi**<br>F.eks. rehabiliteringsbehov fylles av familie/pleiepersonale eller egen trening under tilsyn av en behandler, f.eks. 1-2 timer totalt/månedlig
**TI 2**	**Regelmessige tiltak** for vedlikehold/behandling, f.eks. **hver 1-2 uke**: poliklinisk eller hjemmebasert behandling
**TI 3**	**Behov for hyppige tiltak** bestående av flere møter hver uke

5. BEHOV FOR YRKES-/UTDANNINGSRETTET STØTTE

**VR 0**	**Ikke behov** for yrkes-/utdanningsmessig støtte
**VR 1**	**Behov for yrkesvurdering /-rådgivning** eller **erklæring om utdanningsbehov**
**VR 2**	**Behov for løpende yrkes-/utdanningsrettet støtte,** f.eks. arbeidsmarkedstiltak
**VR 3**	**Behov for formell yrkes-/utdanningsrettet rehabilitering,** f.eks. yrkesforberedelse, omskolering, tilrettelagt arbeid

6. SOSIAL RÅDGIVNING OG OPPFØLGING I KOMMUNE– **støtte/intervensjon for koordinering av omsorg/tjenester**

**S 0**	**Ikke behov** for sosial rådgiving og oppfølging
**S 1**	Behov for **intervensjon av og til eller som er tilgjengelig for råd** ved behov f.eks. kontakt **2-3 x i året**
**S 2**	Behov for **regelmessig intervensjon eller kontakt,** f.eks. **hver eller annenhver måned**
**S 3**	Behov for **hyppig intervensjon eller kontakt,** f.eks. **hver eller annenhver uke**

7. BEHOV FOR STØTTE TIL FAMILIE/OMSORGSPERSON OG AVLASTNING

**7a: Støtte til omsorgsperson i familien**	
**FC 0**	**Ikke behov** for støtte til omsorgsperson i familien
**FC 1**	**Behov for vurdering av behov** hos familie/omsorgsperson
**FC 2**	**Behov for tidsbegrenset støtte til familie/omsorgsperson,** f.eks. for ferdighetstrening
**FC 3**	**Behov for løpende støtte til familie/omsorgsperson,** f.eks. for følelsesmessig støtte

**7b. Avlastning - institusjon og dagsenter:**		
**AVLASTNING I INSTITUSJON**		
**RR 0**	**Ikke behov** for avlastning i institusjon	**Type avlastning**<br>☐ Hjemmebasert midlertidig omsorg fra avlaster i hjemmet<br>☐ Omsorgsbolig/ avlastningsbolig<br>☐ Sykehjem<br>☐ Spesialisert sykehjem<br>☐ Hospice<br>☐ Annet
**RR 1**	Behov for **avlastning i institusjon av og til –** f.eks. i ferier etc.	
**RR 2**	Behov for **regelmessig planlagt avlastning i institusjon,** men ikke svært hyppig (f.eks. 1-2 uker for hver 6 måneder)	
**RR 3**	Behov for **hyppig planlagt avlastning i institusjon** (f.eks. hver 4-6 uker) OG/ELLER **støtte tilgjengelig under kriser**	
**OMSORG PÅ DAGTID**		
**RD 0**	**Ikke behov** for omsorg på dagtid	**Type omsorg på dagtid**<br>☐ Lokalt dagsenter<br>☐ Spesialisert dagsenter<br>☐ Hospice
**RD 1**	**Omsorg på dagtid av og til** – 1-2 dager i uken	
**RD 2**	**Hyppig omsorg på dagtid** – 3-5 dager i uken	

8. BEHOV FOR VERGE –

**AD 0**	**Ikke behov** for verge
**AD 1**	Behov for **vurdering av samtykkekompetanse**
**AD 2**	Behov for **verge eller legalfullmakt**

9. HJELPEMIDLER – f.eks. sittemøbler, tekniske hjelpemidler, pusteutstyr

**E 0**	**Ikke behov for hjelpemidler**	**Typer utstyr**<br>□ Enkel heis/løfteutstyr<br>□ Sittemøbler/rullestol<br>□ Støtte til å stå oppreist<br>□ Elektroniske hjelpemidler<br>□ Kommunikasjonshjelpemidler<br>□ Pusteutstyr<br>□ Annet
**E 1**	**Behov for grunnleggende utstyr** (f.eks. fra hjelpemiddelsentral, som kjøkkenutstyr, toalettstol, seng, sengebøyle osv.)	
**E 2**	**Behov for spesialutstyr –** utstyr som krever faglig vurdering og anskaffelse (f.eks. sittemøbler, hjelp til å stå oppreist)	
**E 3**	**Behov for høyt spesialisert utstyr –** skreddersydd utstyr som må rekvireres/det må søkes om (f.eks. miljøkontroll, kommunikasjons-hjelpemidler, pusteutstyr)	

10. BOLIGBEHOV

**AC 0**	**Ikke behov for spesiell bolig**
**AC 1**	**Begrenset boligtilbud** (f.eks. behov for bolig i første etasje eller tilgang til heis)
**AC 2**	**Behov for delvis tilpasset bolig** (f.eks. håndrekkverk, ramper osv.)
**AC 3**	**Behov for fullt tilpasset bolig** (f.eks. fullt tilgjengelig for rullestol)
**OMSORGS- OG INSTITUSJONSBOLIG**	
**AC 4**	**Behov for omsorgsbolig** med lavt nivå av tilsyn
**AC 5**	**Behov for overvåket boligenhet, f.eks. hjem for en liten gruppe**
**AC 6**	**Behov for hjemmesykepleie i egen bolig eller omsorgsbolig**
**AC 7**	**Behov for omsorg i sykehjem**
**AC 8**	**Behov for omsorg i spesialisert sykehjem**
**AC9**	**Behov for omsorg i hospice**

Del B: Tjenester som gis

Del B er utformet for å gjenspeile Del A,

men registrerer hva denne personen faktisk får –

og kartlegger dermed <u>udekket behov</u>

DEL B – Bistand som gis

**PASIENTINFORMASJON**		
**Navn:**	**NHS-nr:**	**Dato for skåring:...../...../.......**

For hver underskala, sett en ring rundt det høyeste nivået som passer

1. MEDISINSKE OPPFØLGINGSTJENESTER – intervensjon fra en lege for **utredning, tilsyn eller behandling.**

- Medisinsk spesialistbehandling omfatter alle medisinske spesialiteter.

**M 0**	**Fastlege, av og til –** ingen regelmessig kontakt – oppsøker fastlegen selv ved behov	Type med. omsorg<br>☐ Nevrologi<br>☐ Rehab. medisin<br>☐ Nevropsykiatri<br>☐ Palliativ medisin<br>☐ Annet<br>- Nevrokirurgi<br>- Ortopedi<br>- Psykiatri
**M 1**	**Fastlege, aktivt tilsyn -** regelmessig tilsyn/behandling, bare hos fastlegen	
**M 2**	**Spesialiststøtte på lavt nivå** - f.eks. for hovedsaklig stabil tilstand (f.eks. 1-2 besøk per år)<br>Løpende tilsyn/behandling hos fastlege med råd/vurdering hos spesialist av og til	
**M 3**	**Aktiv intervensjon fra medisinsk spesialist** f.eks. for foranderlig/ ustabil tilstand eller for uavklarte symptomer (f.eks. ≥3 besøk per år)<br>Undersøkelse eller behandling som krever hyppig kontakt med spesialisert medisinsk team	

2: SPESIALSYKEPLEIETJENESTER – **intervensjon fra spesial- og/eller spesialtrent sykepleiepersonale,** f.eks. helsesøster eller spesialsykepleier (f.eks. for sårpleie, håndtering av blære-/tarmfunksjonen / tilsyn med medisinbruk/ spesialisert rådgivning / støtte / veiledning).

**N 0**	**Ingen tjenester for** spesialsykepleie	Typer sykepleie<br>☐ Hjemmesykepleier<br>☐ Spesialsykepleier<br>☐ Nevrologi<br>☐ Psykiatri<br>☐ Palliativ omsorg<br>☐ Annet<br>- Sårsykepleier<br>- Stomisykepleier
**N 1**	**Intervensjon av og til** fra en **spesial- eller spesialtrent sykepleier** (f.eks. **månedlig eller sjeldnere)**	
**N 2**	**Regelmessig intervensjon** fra en **spesial- eller spesialtrent sykepleier** f.eks. hver **1-2 uker**	
**N 3**	**Hyppig intervensjon** fra en **spesial- eller spesialtrent sykepleier, hver dag**, eller **flere ganger i uken**	

3. PERSONLIG PLEIE - I og rundt hjemmet

3a: Antall pleiere: til rådighet for hjelp med grunnleggende egenpleie

**CN 0**	**Ingen pleiere** for grunnleggende omsorgsaktiviteter	Hvem yter denne hjelpen?:<br>☐ Uformell familieomsorg<br>☐ Formelle betalte pleiere<br>☐ Andre
**CN 1**	**1 pleier**	
**CN 2**	**2 pleiere**	

3b: Omsorgsfrekvens: Omsorgsfrekvens for hjelp med grunnleggende egenpleie, inkludert trygghet

**CF 0**	**Ikke ytelse** av hjelp til egenpleie
**CF 1**	**Omsorgsbesøk av og til** – sjeldnere enn daglig for hjelp med egenpleie eller utvidede daglige aktiviteter
**CF 2**	**Omsorgsbesøk én gang hver dag,** 1-2 timer

**CF 3**	**2-3 omsorgsbesøk hver dag** – (eller 3-6 timer omsorg hver dag totalt) - ingen omsorg om natten
**CF 4**	**Avlaster i hjemmet eller full dags omsorgspakke** - >6 timer
**CF 5**	**1-til-1 omsorg gjennom hele dagen** OG/ELLER **våkende nattlig omsorg**

3c: Personlig assistent/tilrettelegger **Bistand til deltagelse i lokale aktiviteter på dagtid**

**PA 0**	**Ikke ytelse** av bistand til lokale aktiviteter	Type:<br>☐ Brukerstyrt assistent<br>☐ Støttekontakt<br>☐ Treningskontakt
**PA 1**	**Ytelse av bistand av og til** – 1-2 dager i uken	
**PA 2**	**Ytelse av hyppig bistand** – 3-5 dager i uken	
**PA 3**	**Ytelse av daglig bistand** – 6-7 dager i uken	

4. BEHANDLINGSTJENESTER **– inkludert poliklinisk, aktivitetsbasert og yrkesmessig rehabilitering**

Antall faggrupper: - aktivt involvert i behandling (dvs. minst 1 time i måneden)

**TD 0**	**0**	**Kryss av de faggrupper som er involvert:**<br>☐ Fysioterapi<br>☐ Ergoterapi<br>☐ Logopedi<br>☐ Dietetiker/ ernæringsfysiolog<br>☐ Ortopedi/protese	☐ Psykologi<br>☐ Fam.terapeut/ rådgiver<br>☐ Psykiatri<br>☐ Annet
**TD 1**	**Bare én enkelt faggruppe**		
**TD 2**	**Enkeltstående faggrupper,** ikke koordinert		
**TD 3**	**Koordinert tverrfaglig team**		

Terapiintensitet: - Generell intensitet i de faglige behandlingstiltakene

**TI 0**	**Ingen behandlingstiltak** (eller <1 time i måneden)
**TI 1**	**Periodevis vurdering eller vedlikeholdsprogram** –- omtrent **1-2 timer i måneden** totalt – **ELLER** går **bare** til **gruppeterapi**
**TI 2**	**Regelmessige tiltak** for vedlikehold/behandling, f.eks. **hver 1-2 uke**: Poliklinisk eller hjemmebasert behandling
**TI 3**	**Hyppige tiltak** bestående av **flere møter hver uke** (kan være med ulike faggrupper)

5. TJENESTER FOR YRKES-/UTDANNINGSMESSIG STØTTE

**VR 0**	**Ingen ytelse** av yrkes-/utdanningsrettet støtte
**VR 1**	**Har fått/får yrkes-/utdanningsvurdering/rådgivning** eller **erklæring om utdanningsbehov**
**VR 2**	**Får løpende yrkes-/utdanningsrettet støtte**, f.eks. arbeidsmarkedstiltak
**VR 3**	**Får formell yrkes-/utdanningsrettet rehabilitering**, f.eks. yrkesforberedelse, omskolering, tilrettelagt arbeid

6. SOSIAL RÅDGIVNING OG OPPFØLGING –- **støtte/intervensjon for koordinering av omsorg/tjenester**

**S 0**	**Ingen tjenester** for sosial rådgivning eller oppfølging – **eller svært inkonsistent** (dvs. i praksis ingen)
**S 1**	**Intervensjon eller kontakt for rådgivning av og til** ved behov f.eks. **2-3 ganger i året**
**S 2**	**Regelmessig intervensjon eller kontakt,** f.eks. **hver eller annenhver måned**
**S 3**	**Hyppig intervensjon eller kontakt,** f.eks. **hver eller annenhver uke**

7. STØTTETJENESTER TIL FAMILIE/OMSORGSPERSON/AVLASTNING

7a: Støtte til omsorgsperson i familien	
**FC 0**	**Ingen støttetjenester** til familie/omsorgsperson
**FC 1**	**Har fått/får vurdering av behov** hos familie / omsorgsperson
**FC 2**	**Har fått/får støtte til familie/omsorgsperson**, f.eks. for ferdighetstrening
**FC 3**	**Får løpende støtte til familie/omsorgsperson,** f.eks. for følelsesmessig støtte

7b. Avlastning - institusjon og dagsenter:		
**AVLASTNING I INSTITUSJON**		
**RR 0**	**Ingen tjenester** for avlastning i institusjon	**Type avlastning**<br>☐ Hjemmebasert midlertidig omsorg fra avlaster i hjemmet<br>☐ Omsorgsbolig/ avlastningsbolig<br>☐ Sykehjem<br>☐ Spesialisert sykehjem<br>☐ Hospice<br>☐ Annet
**RR 1**	**Tjenester for avlastning i institusjon av og til –** f.eks. i ferier etc.	
**RR 2**	**Tjenester for regelmessig planlagt avlastning i institusjon**, men ikke svært hyppig (f.eks. 1-2 uker for hver 6 måneder)	
**RR 3**	**Hyppig planlagt avlastning i institusjon** (f.eks. hver 4-6 uke) OG/ELLER **støtte tilgjengelig under kriser**	
**OMSORG PÅ DAGTID**		
**RD 0**	**Ingen tjenester** for omsorg på dagtid	**Type omsorg på dagtid**<br>☐ Lokalt dagsenter<br>☐ Spesialisert dagsenter<br>☐ Hospice
**RD 1**	**Tjenester for omsorg på dagtid av og til** – 1-2 dager i uken	
**RD 2**	**Hyppige tjenester for omsorg på dagtid** – 3-5 dager i uken	

8. YTELSE AV VERGETJENESTER

**AD 0**	**Ingen ytelse** av vergetjenester
**AD 1**	**Har fått/får vurdering av samtykkekompetanse**
**AD 2**	**Har en verge eller legalfullmakt**

9. SPESIALUTSTYR – f.eks. spesielle sittemøbler, tekniske hjelpemidler, pusteutstyr

**E 0**	**Ingen hjelpemidler / utilstrekkelige tjenester**	**Typer utstyr**<br>☐ Enkel heis/løfteutstyr<br>☐ Sittemøbler/rullestol<br>☐ Støtte til å stå oppreist<br>☐ Elektroniske hjelpemidler<br>☐ Kommunikasjonshjelpemidler<br>☐ Pusteutstyr<br>☐ Annet
**E 1**	**Ytelse av grunnleggende utstyr** (f.eks. fra hjelpemiddelsentral, som kjøkkenutstyr, toalettstol, seng, sengebøyle osv.)	
**E 2**	**Ytelse av spesialutstyr –** utstyr som krever faglig vurdering og anskaffelse (f.eks. sittemøbler, hjelp til å stå oppreist)	
**E 3**	**Ytelse av høyt spesialisert utstyr –** skreddersydd utstyr som må rekvireres/det må søkes om (f.eks. miljøkontroll, kommunikasjons-hjelpemidler, pusteutstyr)	

10. BOLIGTILBUD

**AC 0**	**Ikke tilbud om spesiell bolig**
**AC 1**	**Begrenset boligtilbud imøtekommet** (f.eks. behov for bolig i første etasje eller tilgang til heis)
**AC 2**	**Har delvis tilpasset bolig** (f.eks. håndrekkverk, ramper osv.)
**AC 3**	**Har fullt tilpasset bolig** (f.eks. fullt tilgjengelig for rullestol)
**OMSORGS- OG INSTITUSJONSBOLIG**	
**AC 4**	**Har omsorgsbolig** med lavt nivå av tilsyn
**AC 5**	**Har overvåket bolig, f.eks. hjem for en liten gruppe**
**AC 6**	**Har egen bolig eller omsorgsbolig med hjemmesykepleie**
**AC 7**	**Har omsorg i sykehjem**
**AC 8**	**Har omsorg i spesialisert sykehjem**
**AC9**	**Har omsorg i hospice**

Validering av NPCS

En foreløpig validering av NPCS er blitt foretatt, og funnene ble presentert på *World Congress in Neuro-Rehabilitation* i Melbourne, mai 2012.

En første evaluering i en kohort på 423 pasienter utskrevet etter innleggelse i spesialiserte rehabiliteringsinstitusjoner over hele London-regionen viste at NPCS var enkelt å forstå og fylle ut, både for klinikere og pasienter.

- Behov ble rangert av de behandlende klinikerne ved utskriving.
- Pasientene og/eller deres omsorgspersoner i familien registrerte omfanget av tjenestene i forhold til behov i et postalt egenrapporteringsskjema (med oppfølgende telefonintervju der det var nødvendig).

En eksplorerende faktoranalyse viste to hovedfaktorer, som gjenspeilte behov for *'Helse og personlig omsorg'* og *'Sosial og familiebasert støtte'*.

- Skalaens totale reliabilitet var fremragende, med Cronbachs α=0,94.
- Test/retest-reliabiliteten for egenrapportering var oppmuntrende, med ICCer for de seks underskalaene som varierte fra 0,61 til 0,85.
- Overensstemmelsen mellom spørsmålene, målt med den kvadratisk vektede Cohens kappa, varierte fra 0,47 til 0,93.

(Lim inn lenke til plakaten her – WCNR-plakat nr. 49 (pdf) – faktorstruktur)

Bruk av NPCS i den samme pasientserien viste betydelige avvik mellom behov og ytelser, spesielt med henblikk på løpende sosial rehabilitering, hjelpemidler og sosial støtte.

- Til sammenligning ble behov for medisinsk oppfølging, sykepleie og personlig pleie relativt godt dekket.
- Ytelse av støtte til personlig pleie utover nivået på forutsette behov antydet en reduksjon i uavhengighet for noen pasienter etter utskriving, muligens som et resultat av manglende dekning av behov for rehabilitering og sosial støtte.

(Lim inn lenke til plakaten her – WCNR-plakat nr. 48 (pdf) – udekkede behov)

En formell psykometrisk evaluering, inkludert en vurdering av reliabilitet mellom ulike bedømmere, ble innsendt for publisering i desember 2012.

Å lage en kostnadsalgoritme innenfor NPCS

Skalaen for behovs- og ytelseskompleksitet (NPCS) gir en ordinalskala for å beregne dekkede og udekkede behov. Det har blitt utviklet en kostnadsalgoritme for å uttrykke virkningen av dekkede og udekkede behov direkte i kostnader.

- Data innsamlet ved bruk av Client Service Receipt Inventory (CSRI) ga informasjon om antallet og varigheten av kontaktene for hver type av tjeneste.
- Disse dataene ble analysert innenfor punktene i NPCS for å avlede et sett av kostnadsantagelser for hvert skåringsnivå.
- Der hvor dataene fra CSRI var utilstrekkelige, ble det gjort intuitive antagelser basert på klinisk erfaring, som deretter ble testet innenfor en faggruppe av klinikere med erfaring i

planlegging og ytelse av lokale tjenester.

Kostnadene ble regnet ut med referanse til Curtis, 201167, og justert der det var nødvendig for å gjenspeile kostnadene ved spesialisert pleie (med nyttige personlige meddelelser fra Lesley Curtis). Enhetskostnader for 2011 ble brukt, siden disse dataene vil bli brukt prospektivt, i motsetning til den retrospektive anvendelsen av kostnader for 2010 for data fra CSRI, som gjenspeilte det året da tjenestene ble mottatt av deltakerne i studien.

(Kostnadsalgoritme for NPCS)

Referanser

Turner-Stokes L, Siegert RJ. *The Needs and Provision Complexity Scale: factor structure and repeatability.*
Poster Presentation. 7th World Congress in Neurorehabilitation; 2012; Melbourne.
Abstact published in *Neurorehabilitation and Neural Repair* 2012; 26(6): 695-804 (Poster 48)

Turner-Stokes L, Siegert RJ. *The Needs and Provision Complexity Scale: measuring met and unmet needs in the community for patients with complex neurological disabilities.*
Poster Presentation. 7th World Congress in Neurorehabilitation; Melbourne. May 2012
Neurorehabilitation and Neural Repair 2012; 26(6): 695-804 (Poster 49)
