# Supplementary material for: Validity of the Norwegian Version of the Needs and Provision Complexity Scale (NPCS) in Patients with Traumatic Brain Injury and Atraumatic Subarachnoid Hemorrhage
Source: J Clin Med. 2024 Jan 28;13(3):752. doi: 10.3390/jcm13030752 (PMC10856215; doi:10.3390/jcm13030752)
Supplement: Supplementary file 1 [file jcm-13-00752-s001.zip › Supplementary Material File S3 NPCS_patient version 2.0_Norwegian.pdf]

King's College London
University of London

The North West London Hospitals NHS Trust

# Skala for behovs- og tjenestekompleksitet ved kroniske nevrologiske sykdommer (NPCS)

# Versjon for pasienter

Skjemaet kan brukes og kopieres fritt,

***men må ha referanse til opphavspersonene ved bruk i publikasjoner***

**For mer informasjon og råd, ta kontakt med:**

Professor Lynne Turner-Stokes DM FRCP
Herbert Dunhill Chair of Rehabilitation, King's College London.

Norsk versjon 2.0 - 2017

# NPCS: BEHOV FOR OMSORG OG STØTTE

**Dagens dato: Dag ☐☐ Måned ☐☐ År ☐☐☐☐**

## Nåværende rehabiliterings- og støttetjenester

- Vi vil gjerne vite noe om de omsorgs- og støttetjenestene du mottar nå.
- Vi vil spørre deg om 12 områder der du vil kunne få støtte fra enten helsetjenesten eller sosialtjenesten.
- Vi er også interessert i om hvorvidt du mener at denne støtten gis i riktig omfang for øyeblikket, eller om du synes at du trenger mer støtte eller mindre støtte for at du skal være fornøyd og ha et godt liv.

(NB til intervjueren – hvis omsorg ikke gis i riktig omfang, prøv å fastslå hva de mener at de trenger (skriv ned med deres egne ord), og pass dette inn på det mest relevante nivået i skjemaet)

## For hvert av spørsmålene under vil vi gjerne vite noe om:

1. Omfanget av omsorg og støtte du har fått **i løpet av de siste seks månedene**.
2. Hvorvidt denne omsorgen og støtten gis i riktig omfang, eller hvorvidt du mener at du ville ha nytte av mer eller mindre omsorg enn det nivået du har mottatt.

Hvis mer enn ett svaralternativ passer, velger du det som står lengst ned på listen

**For eksempel:**
Hvis du er under regelmessig oppfølging fra fastlegen din, og ble undersøkt i løpet av de siste seks månedene,
men også trenger tilsyn av en spesialist av og til,
vil du merke av på denne måten:

| | Ja | Nei |
|---|---|---|
| Får du regelmessig medisinsk oppfølging? | ☒ | ☐ |

Hvis 'ja':

| **Trenger medisinsk tilsyn/tiltak:** | **Kryss av én** |
|---|---|
| a) Regelmessige besøk hos fastlege for tilsyn/behandling | ☐ |
| b) Trenger av og til råd/tilsyn fra spesialist (f.eks. 1-2 besøk i året) | ☒ |
| c) Trenger regelmessig behandling hos spesialist (f.eks. tre eller flere besøk i året) | ☐ |

NPCS engelsk versjon 6; Turner Stokes et al. NPCS norsk versjon 1.0; Akasie språktjeneste, Frich og van Walsem.
NPCS norsk versjon 2.0; Forslund et al.

**Denne delen handler om hvor mye medisinsk oppfølging og sykepleie du mottar**

## 1. MEDISINSK OPPFØLGING

Vi ønsker å vite noe om **oppfølgingen du har mottatt fra en lege (fastlege eller spesialist) for utredning, tilsyn eller behandling**

- Bidrag fra spesialist kan være innenfor alle medisinske spesialiteter

| | Ja | Nei |
|---|---|---|
| Får du regelmessig **medisinsk oppfølging?** | ☐ | ☐ |

Hvis 'ja':

| **Dette omfatter:** | Flere kryss mulig |
|---|---|
| a) Regelmessige besøk hos fastlege for tilsyn/behandling | ☐ |
| b) Trenger av og til råd/undersøkelse hos spesialist (f.eks. 1-2 besøk i året) | ☐ |
| c) Trenger regelmessig behandling hos spesialist (f.eks. tre eller flere besøk i året) | ☐ |

| **Hvilke(n) type(r) spesialist?** | Flere kryss mulig |
|---|---|
| Nevrolog | ☐ |
| Lege som er spesialist i rehabilitering | ☐ |
| Psykiater | ☐ |
| Lege som er spesialist innen palliativ medisin | ☐ |
| Annet, vennligst spesifiser: ………………………………………… (f.eks. nevrokirurg, ortoped) | ☐ |

## 2. OPPFØLGING FRA SPESIALSYKEPLEIER

Vi ønsker å få vite hvilket **nivå av støtte / intervensjon du har fått fra spesialsykepleiere/spesialtrenede sykepleiere.** F.eks. for sårpleie, håndtering av blære-/tarmfunksjonen / tilsyn med medisinbruk / spesialisert rådgivning / støtte / veiledning)

| | Ja | Nei |
|---|---|---|
| Har du fått **støtte av en spesialsykepleier/spesialtrenede sykepleiere** i løpet av de siste seks månedene: | ☐ | ☐ |

Hvis 'ja':

| **Har fått støtte:** | Kryss av én |
|---|---|
| a) Av og til (f.eks. én gang i måneden eller sjeldnere) | ☐ |
| b) Regelmessig (f.eks. hver eller annenhver uke) | ☐ |
| c) Ofte (f.eks. hver dag eller flere ganger i uken) | ☐ |
| d) 2-12 timer hver dag | ☐ |
| e) 12-24 timer hver dag | ☐ |

| **Hvilke(n) type(r) sykepleiere?** | Flere kryss mulig |
|---|---|
| Hjemmesykepleier | ☐ |
| Spesialsykepleier i: | |
| a) Nevrologi | ☐ |
| b) Psykiatri | ☐ |
| c) Palliativ omsorg | ☐ |
| d) Annet, vennligst spesifiser: ……………………………………………………………(f.eks. sårsykepleier, stomisykepleier) | ☐ |

| **Til spørsmål 1-2: Er dette riktig omfang medisinsk oppfølging/sykepleie for deg?** | |
|---|---|
| For lite | ☐ |
| Passe | ☐ |
| For mye | ☐ |
| **Hvis det ikke er riktig omfang, hva trenger du?** ……………………………………………………………………………………… ……………………………………………………………………………………… ……………………………………………………………………………………… | |

**Denne delen handler om den hjelpen du får til aktiviteter i hjemmet og i lokalmiljøet**

### 3. HJELP MED GRUNNLEGGENDE EGENPLEIE I HJEMMET

Vi ønsker å vite noe om **nivået på den hjelpen du får til grunnleggende egenpleie** i og rundt hjemmet ditt.

(**Egenpleie** omfatter det å kle på seg, dusje/bade, gå på toalettet, spise og lage mat)

| | **Ja** | **Nei** |
|---|---|---|
| Har du fått **hjelp til grunnleggende egenpleie** i løpet av de siste seks månedene? | ☐ | ☐ |

| Hvis 'ja':<br>**Fikk du hjelp:** | Kryss av én |
|---|---|
| a) Fra **1 pleier** om gangen | ☐ |
| b) Fra **2 eller flere pleiere** om gangen | ☐ |

| **Hvis du har fått hjelp til grunnleggende egenpleie:**<br><br>**Hvem ga denne hjelpen?** | Flere kryss mulig |
|---|---|
| Familiemedlem(mer) | ☐ |
| Betalt(e) pleier(e) | ☐ |
| Andre, vennligst spesifiser: ………………………………………………………………… | ☐ |

| **Hvor ofte ble denne hjelpen gitt?** | Kryss av én |
|---|---|
| **Av og til** - men ikke hver dag | ☐ |
| **Én gang om dagen** | ☐ |
| **2-3 ganger hver dag** - men ikke om natten | ☐ |
| **For det meste av tiden,** på grunn av hyppige eller uforutsigbare omsorgsbehov | ☐ |
| **Konstant overvåking** og/eller **hjelp flere ganger hver natt** | ☐ |

## 4. HJELP TIL SOSIALE AKTIVITETER I LOKALMILJØET

Vi ønsker å vite hvor ofte du får hjelp til å delta i aktiviteter i lokalmiljøet av støttekontakt, treningskontakt eller brukerstyrt assistent.

F.eks. fritid, arbeid eller sosiale sammenkomster

| | **Ja** | **Nei** |
|---|---|---|
| Har du fått **hjelp til aktiviteter i lokalmiljøet** de siste seks månedene? | ☐ | ☐ |

| Hvis 'ja':<br>**Du fikk hjelp:** | Kryss av én |
|---|---|
| a) Av og til (1-2 dager i uken eller mindre) | ☐ |
| b) Regelmessig (3-5 dager i uken) | ☐ |
| c) Ofte (6-7 dager i uken) | ☐ |

| **Til spørsmål 3-4: Er dette riktig omfang av personlig omsorg for deg?** | |
|---|---|
| For lite | ☐ |
| Passe | ☐ |
| For mye | ☐ |

**Hvis det ikke er riktig omfang, hva trenger du?**

……………………………………………………………………………………………………………………

……………………………………………………………………………………………………………………

……………………………………………………………………………………………………………………

| Denne delen handler om hvilke omfang og typer av behandling/rehabilitering du får<br><br>**5. BEHANDLING**<br>Vi er interessert i hvor mange ulike typer behandlere du oppsøker, og hvor ofte du oppsøker dem.<br>F.eks. fysioterapi, ergoterapi, psykologi, logopedi | | |
|---|---|---|
| | **Ja** | **Nei** |
| Har du fått **behandling** i løpet av de siste seks månedene? | ☐ | ☐ |
| Hvis 'ja':<br>**Har fått behandling fra:** | Kryss av én | |
| a) Bare **én enkelt faggruppe** (f.eks. fysio- eller ergoterapi) | ☐ | |
| b) Mer enn én enkelt faggruppe - men som arbeidet **hver for seg**, ikke som et team | ☐ | |
| c) Mer enn én enkelt faggruppe - **som arbeidet sammen i et koordinert team** | ☐ | |

| **Hvilke typer behandlere oppsøkte du?** | Flere kryss mulig |
|---|---|
| Fysioterapeut | ☐ |
| Ergoterapeut | ☐ |
| Logoped | ☐ |
| Dietetiker/ernæringsfysiolog | ☐ |
| Ortopediingeniør / protesemaker | ☐ |
| Psykolog | ☐ |
| Familieterapeut eller annen rådgiver | ☐ |
| Psykiater | ☐ |
| Andre, vennligst spesifiser:<br>……………………………………………………………………………(f.eks. sosionom, optiker) | ☐ |

| **Hvis du har fått behandling:**<br>**Hvor ofte møtte du behandleren/-e?** | Kryss av én |
|---|---|
| **a) Av og til** (én time i måneden) eller **behandling bare i gruppemøter** | ☐ |
| **b) Regelmessige individuelle møter** - hver eller annenhver uke | ☐ |
| **c) Hyppige individuelle møter** - flere ganger i uken | ☐ |

## 6. STØTTE FOR Å KOMME TILBAKE I ARBEID ELLER UTDANNING

Vi ønsker å få vite hvilken **arbeidsrettet støtte du har fått for å komme tilbake til** fulltids eller deltids**arbeid eller utdanning**.
F.eks. yrkesveiledning for uføre, arbeidsrettet omskolering, arbeidsmarkedstiltak.

| | Ja | Nei |
|---|---|---|
| Har du fått **hjelp/støtte for å komme tilbake i arbeid/utdanning** i løpet av de siste seks månedene? | ☐ | ☐ |

Hvis 'ja':

| **Har fått hjelp:** | Kryss av én |
|---|---|
| a) Bare for arbeidsrelatert/utdanningsmessig vurdering/rådgivning (1-2 møter) | ☐ |
| b) Løpende arbeidsrelatert/utdanningsmessig støtte, f.eks. arbeidsmarkedstiltak | ☐ |
| c) Et formelt yrkesmessig program for arbeids- eller utdanningsrettet støtte, f.eks. yrkesforberedelse, omskolering, tilrettelagt arbeid | ☐ |

### Til spørsmål 5-6: Er omfanget av rehabiliteringen riktig for deg?

| | |
|---|---|
| For lite | ☐ |
| Passe | ☐ |
| For mye | ☐ |

**Hvis det ikke er riktig omfang, hva trenger du?**

.......................................................................................................................................

.......................................................................................................................................

.......................................................................................................................................

### Denne delen handler om omfanget av den sosiale støtten du får.

## 7. SOSIAL RÅDGIVNING OG OPPFØLGING I KOMMUNE

Vi ønsker å vite noe om **hvilken sosial rådgivning og oppfølging** du har fått.
Dette kan f.eks. være i form av en ansvarsgruppe eller koordinator i kommunen.

| | Ja | Nei |
|---|---|---|
| Har du fått **sosial rådgivning eller oppfølging** i løpet av de siste seks månedene? | ☐ | ☐ |

Hvis 'ja':

| **Har fått hjelp/støtte:** | Kryss av én |
|---|---|
| a) Tilgjengelig for råd når det er nødvendig | ☐ |
| b) 1-2 avtaler | ☐ |
| c) 3 eller flere avtaler | ☐ |

| **8. STØTTE TIL FAMILIEN**<br>Vi ønsker å få vite hvilken **støtte som er mottatt for eventuelle omsorgspersoner i familien** | | |
|---|---|---|
| | **Ja** | **Nei** |
| Har du **mottatt støtte for omsorgspersoner i familien** i løpet av de siste seks månedene? | ☐ | ☐ |
| Hvis 'ja':<br>**Mottatt støtte for omsorgspersoner:** | Kryss av én | |
| a) Bare vurdering - for å se hvilken støtte det kunne være behov for | ☐ | |
| b) Noe kortsiktig støtte til omsorgspersoner/familie, f.eks. for opptrening av ferdigheter | ☐ | |
| c) Løpende støtte til omsorgspersoner/familie, f.eks. for følelsesmessig støtte | ☐ | |

**Denne delen handler om avlastning som gis for at omsorgspersoner i familien skal få en pause**

Dette kan skje enten i **en avlastningsbolig eller på et dagsenter**

| **9. AVLASTNING**<br>**A) Vi ønsker å få vite om behov for fulltids omsorg i en avlastningsbolig, f.eks. et sykehjem eller et hospice.** | | |
|---|---|---|
| | **Ja** | **Nei** |
| Har du mottatt **omsorg i en avlastningsbolig** i løpet av de siste seks månedene? | ☐ | ☐ |
| Hvis 'ja':<br>**Har fått omsorg i en avlastningsbolig:** | Kryss av én | |
| a) Én gang | ☐ | |
| b) To ganger | ☐ | |
| c) Tre eller flere ganger | ☐ | |

| **Hvilken type avlastningsbolig?** | Flere kryss mulig |
|---|---|
| Hjemmebasert omsorg ved hjelp av avlaster i hjemmet | ☐ |
| Omsorgsbolig | ☐ |
| Sykehjem | ☐ |
| Spesialisert sykehjem | ☐ |
| Hospice | ☐ |
| Annet, vennligst spesifiser: …………………………………………………………………… | ☐ |

| **B)** Vi ønsker å få vite noe om behov for **omsorg på dagtid**, f.eks. på et dagsenter | | |
|---|---|---|
| | **Ja** | **Nei** |
| Har du fått **omsorg på dagtid** i løpet av de siste seks månedene? | ☐ | ☐ |
| Hvis 'ja':<br>**Har fått omsorg på dagtid:** | Kryss av én | |
| a) Av og til, f.eks. 1-2 dager i uken | ☐ | |
| b) Ofte, f.eks. 3-5 dager i uken | ☐ | |

| **Hvilken type omsorg på dagtid?** | Flere kryss mulig |
|---|---|
| Lokalt dagsenter | ☐ |
| Spesialisert dagsenter | ☐ |
| Hospice | ☐ |
| Annet, vennligst spesifiser:<br>……………………………………………………………………………… | ☐ |

| **10. BEHOV FOR VERGE**<br>En verge er en som kan representere interessene og rettighetene til noen som mangler mental kapasitet til å ta beslutninger på egne vegne.<br>I mange tilfeller vil familien ha denne rollen, men av og til er det behov for en uavhengig oppnevnt verge. | Kryss av én |
|---|---|
| **I løpet av de siste seks månedene:**<br>a) Har det **ikke vært behov for verge** | ☐ |
| b) **Har fått vurdering av mental kapasitet** til å ta beslutninger om omsorg | ☐ |
| c) Mangler mental kapasitet, og **familien gir støtte når det skal tas beslutninger** | ☐ |
| d) Mangler samtykkekompetanse, og **har fått en verge eller legalfullmakt** | ☐ |

| **Til spørsmål 7-10: Er dette riktig omfang av sosial støtte/familiestøtte for deg?** | |
|---|---|
| For lite | ☐ |
| Passe | ☐ |
| For mye | ☐ |
| **Hvis det ikke er riktig omfang, hva trenger du?**<br>…………………………………………………………………………………………………………<br>…………………………………………………………………………………………………………<br>………………………………………………………………………………………………………… | |

**Denne delen handler om hjelpemidler og utstyr, eller tilpasning av boligen.**

## 11. HJELPEMIDLER

Vi vil vite noe om hvilke **hjelpemidler og utstyr** du har.

| | Ja | Nei |
|---|---|---|
| Har du mottatt **hjelpemidler eller utstyr** i løpet av de siste seks månedene? | ☐ | ☐ |

Hvis 'ja':

| **Har mottatt hjelpemidler/utstyr:** | Kryss av én |
|---|---|
| a) Enkelt utstyr som er i fritt salg, f.eks. kjøkkenhjelpemidler, toalettstol, løftebøyle for seng | ☐ |
| b) Spesialutstyr som krever faglig vurdering/anskaffelse (f.eks. spesiell rullestol) | ☐ |
| c) Høyt spesialisert/spesiallaget utstyr som må rekvireres/det må søkes om (f.eks. miljøkontrollsystemer, kommunikasjonshjelpemidler, pusteutstyr) | ☐ |

| **Hvilke(n) type(r) av utstyr?** | Flere kryss mulig |
|---|---|
| Enkel heis/løfteutstyr | ☐ |
| Sittemøbler/rullestol | ☐ |
| Støtte til å stå oppreist (rullator) | ☐ |
| Elektroniske hjelpemidler | ☐ |
| Kommunikasjonshjelpemidler | ☐ |
| Hjelpemidler for pustefunksjon | ☐ |
| Annet, vennligst spesifiser: ……………………………………………………………………………… | ☐ |

## 12. BOLIG

### a) Hvis du KAN bo i ditt eget hjem:

Vi vil gjerne vite dine behov for tilpasning av boligen.

| | Ja | Nei |
|---|---|---|
| Har du en **tilpasset bolig?** | ☐ | ☐ |

Hvis 'ja':

| **Din tilpassede bolig er:** | Kryss av én |
|---|---|
| a) I første etasje eller har tilgang til en pålitelig heis | ☐ |
| b) Noe tilpasset (f.eks. håndrekkverk, ramper) | ☐ |
| c) En fullt tilpasset bolig (f.eks. fullt tilgjengelig for rullestol) | ☐ |

| **ELLER b) Hvis du IKKE KAN bo i ditt eget hjem:**<br>Vi ønsker å få vite om boligen har støttefunksjoner eller om du får omsorg i institusjon. | | |
|---|---|---|
| | **Ja** | **Nei** |
| Bor du i en **bolig med støttefunksjoner eller på en institusjon?** | ☐ | ☐ |
| Hvis 'ja':<br>**Din bolig med støttefunksjoner eller institusjon er en:** | Kryss av én | |
| a) Overvåket boligenhet, f.eks. et bofellesskap for en liten gruppe | ☐ | |
| b) Omsorgsbolig, f.eks. med tilsyn | ☐ | |
| c) Egen bolig med hjemmesykepleie | ☐ | |
| d) Sykehjem | ☐ | |
| e) Spesialisert sykehjem | ☐ | |
| f) Hospice | ☐ | |

| **Til spørsmål 11-12: Er dette riktig omfang av hjelpemidler/boligtilbud for deg?** | |
|---|---|
| For lite | ☐ |
| Passe | ☐ |
| For mye | ☐ |

**Hvis det ikke er riktig omfang, hva trenger du?**

……………………………………………………………………………………………………………………

……………………………………………………………………………………………………………………

……………………………………………………………………………………………………………………

## 13. PRIVATE TJENESTER

Betaler du i dag for noen private rehabiliterings- eller medisinske tjenester?

Sett en ring rundt svaret:

Ja Nei

Hvis ja, hvilke tjenester?

……………………………………………………………………………………………………………………
…………

………………………………………………………………………………………...……………………...……
……………

## 14. VENTELISTE

Står du på venteliste for noen rehabiliterings- eller støttetjenester?

Sett en ring rundt svaret:

Ja Nei

Hvis ja, hvilke tjenester?
…………………………………………………………..……………………………………………………………………
….

……………………………………………………………………………………………………………………………

Når er de forventet å starte? …………..……………………………………………………………………….

…………………………………………………………………………………………………………………………….
